# Chronologically overlapping occurrences of nicotine-induced anxiety- and depression-related behavioral symptoms: effects of anxiolytic and cannabinoid drugs

**DOI:** 10.1186/1471-2202-8-76

**Published:** 2007-09-18

**Authors:** Tamaki Hayase

**Affiliations:** 1 Department of Legal Medicine, Kyoto University, Kyoto 606-8501, Japan

## Abstract

**Background:**

Anxiety and depression are among the most frequently-observed psychiatric symptoms associated with nicotine (NC). In addition to the similarity to other addictive drugs, these NC-induced symptoms are characteristic in that the opposite behavioral effects, i.e. anxiolytic and antidepressant effects, which may reinforce the habitual use of NC, have also been reported. In the present study, the time course of anxiety- and depression-related behavioral alterations was examined in mice. Furthermore, based on the reported similarity in the mechanisms responsible for NC-induced anxiety- and depression-related symptoms, as well as the contribution of brain cannabinoid (CB) receptors to these behavioral symptoms, the effects of anxiolytics and CB receptor ligands (CBs) against these behavioral symptoms were investigated.

**Results:**

Repeated subcutaneous NC treatments (0.3 mg/kg, 4 days), compared with a single treatment (0.5 mg/kg), caused both prolonged anxiogenic effects in the elevated plus-maze test, and prolonged depressive effects in the forced swimming test, even at 120 min time point after the last NC treatment. A transient anxiolytic preference for open arms was also observed in the elevated plus-maze test. Among the anxiolytics and CBs, the serotonin 1A (5-HT1A) antagonist WAY 100135 and the endogenous mixed CB agonist/antagonist virodhamine (VD), when administered intraperitoneally before each NC treatment, provided the strongest antagonistic effects against the anxiety-related symptoms. However, against the depression-related symptoms, only VD provided significant antagonistic effects in both single and repeated treatment groups.

**Conclusion:**

The present results support the presence of a chronological overlap of NC-induced anxiety- and depression-related behavioral symptoms, and the contribution of brain CB receptors to these behavioral symptoms. The repeated NC-induced prolongation of these behavioral symptoms and the early transient anxiolytic behavioral alterations support an increased possibility of the habitual use of NC. Furthermore, based on the antagonistic effects of VD, one can predict that the characteristic effects on brain CB receptors as a mixed CB agonist/antagonist contributed to its therapeutic effects as both an anxiolytic and an antidepressant.

## Background

Anxiety and depression are among the most frequently-observed psychiatric effects associated with nicotine (NC) in tobacco smoking [1-4], similar to cases using well-known addictive psychostimulants such as cocaine [5,6]. These NC-related symptoms have been reported as direct effects of NC, which disappear following smoking cessation [2,4] or as withdrawal symptoms [1,3]. In experimental models, similar to the anxiety- and depression-related behavioral symptoms from other causes, brain GABAergic and serotonin (5-HT) neurons contribute to the anxiogenic effects of NC [7-9], whereas brain monoamine neurons contribute to its depressive effects [10]. However, since the distribution of brain nicotinic acetylcholine receptors (nAChRs), the primary targets of NC, is widespread and functional interactions between the above indicated anxiety- and depression-related neurons and nAChRs have been demonstrated [11-13], different mechanisms mediating anxiety- and depression-related behaviors as compared to behaviors caused by the other addictive drugs can be predicted. For example, NC also causes anxiolytic and antidepressant effects in both humans and animals, depending on dose, time after use, and previous exposure to NC [14-17], and these effects seem to reinforce the habitual use of NC. Furthermore, because of the overlapped distributions of nAChRs and the targets of other abused drugs such as DA neurons, the targets of addictive psychostimulants, and the similarity of the effects of NC to those of other addictive drugs [18,19], NC is thought to play a role as a trigger for other abused drugs [20].

The anxiogenic effects of NC are, like the anxiety-related behavioral symptoms from other causes, attenuated by benzodiazepine (BZ)- and 5-HT-related anxiolytics in several animal models [7-9]. However, based on the above-explained characteristic pharmacological effects of NC (i.e. the induction of contrary behavioral symptoms depending on the treatment conditions) and the widespread distributions of nAChRs, it is possible that the role of these BZ- and 5-HT-related drugs as anxiolytics may be modified in NC treatment groups. Furthermore, although some contributions of GABAergic and 5-HT neurons, the targets of BZ- and 5-HT-related anxiolytics, to depression-related behavioral symptoms have also been suggested [21,22], the effects of these drugs against the NC-induced depression-related behavioral symptoms have not been studied. Recently, functional interactions between brain cannabinoid (CB) receptors and nAChRs have been demonstrated, and modifications of the behavioral effects of NC by CB receptor ligands (cannabinoids: CBs), e.g. attenuation of NC-induced anxiety-related behavioral symptoms by a CB agonist, have been elucidated [23-25]. Functional interactions between brain CB receptors and receptors related to anxiety and/or depression (e.g. modifications of GABAergic, serotonergic, and monoaminergic neurotransmission by CBs, etc.) have also been demonstrated [26-28]. Furthermore, several endogenous CBs were discovered, and their potential therapeutic uses, including anxiolytic and antidepressant effects reported for endogenous agonists such as anandamide, have been suggested [28-30].

In the present study, based on the predicted peculiarity of the behavioral effects of NC, the time course of anxiety-related (elevated plus-maze test) and depression-related (forced swimming test) behavioral alterations were examined in both single and repeated treatment groups of mice, and the characteristics of the appearance of these behavioral symptoms, which may be correlated with the habitual use of NC, were investigated. Furthermore, one of the objectives of the present study was to examine the effects of both conventional BZ- and 5-HT-related anxiolytics and CBs, some of which can function as both anxiolytics and antidepressants, against the NC-induced anxiety- and depression-related behavioral symptoms, and to investigate the possible use of these drugs as both anxiolytics and antidepressants.

## Results

### Time course of NC-induced anxiety-related behavioral alterations

**Figure 1:**
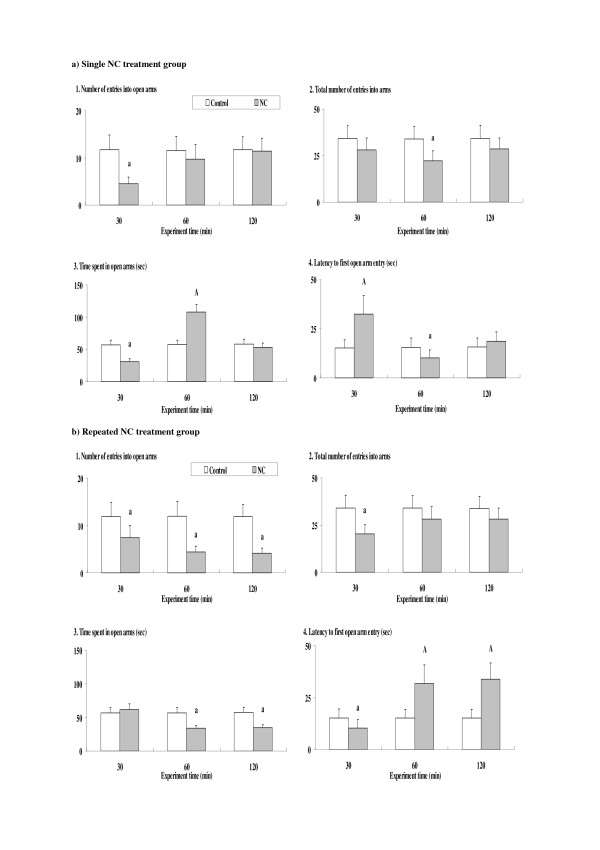
**Time courses of nicotine (NC)-induced anxiety-related behavioral alterations in the elevated plus-maze test**. The parameter values at 30, 60 and 120 min time points after the NC treatment are shown for the single (a) and repeated (b) NC treatment groups. Data represent means ± SD (n = 7 for each group). A, a: significant (p < 0.05) increase (A) or attenuation (a) as compared to the control group.

For the single NC (0.5 mg/kg) treatment group (Fig. 1a), at 30 min time point, behavioral alterations derived from an anxiety-related attenuated preference for open arms (significantly attenuated number of entries into and time spent in open arms, and increased latency to first open arm entry) were observed for each parameter value in the elevated plus-maze test. On the other hand, at 60 min time point, there was a significantly increased amount of time spent in open arms and an attenuated latency to first open arm entry, suggesting attenuations in anxiety-related behavioral alterations and anxiolytic effects of NC. With respect to the total number of entries into arms, there was no significant difference as compared to the control group at 30 min time point. However, at 60 min time point, a significant attenuation in the total number of entries into arms, indicating an increased ratio of the number of entries into open arms and suggesting anxiolytic effects of NC, was observed. All of the altered parameter values returned to the control levels at 120 time point.

For the repeated NC (0.3 mg/kg, 4 days) treatment group (Fig. 1b), at 30 min time point, a tendency towards an increased amount of time spent in open arms and a significantly attenuated latency to first open arm entry, suggesting anxiolytic effects of NC, was observed. On the other hand, at 60 min time point, behavioral alterations derived from an anxiety-related preference for closed arms were observed for each parameter value. For the total number of entries into arms, a significant attenuation, suggesting anxiolytic effects of NC, was observed at 30 min time point. However, at 60 min time point, there was no significant difference as compared to the control group. At 120 min time point, the anxiety-related attenuated preference for open arms persisted.

In both single and repeated treatment groups, the ANOVA analyses revealed significant main effects of NC treatment for each parameter value [see Additional file 1]. Furthermore, significant main effects of test time and interactions between NC treatment and test time were also observed for some parameter values, which indicated some time-dependent changes.

### Time course of NC-induced depression-related behavioral alterations

**Figure 2:**
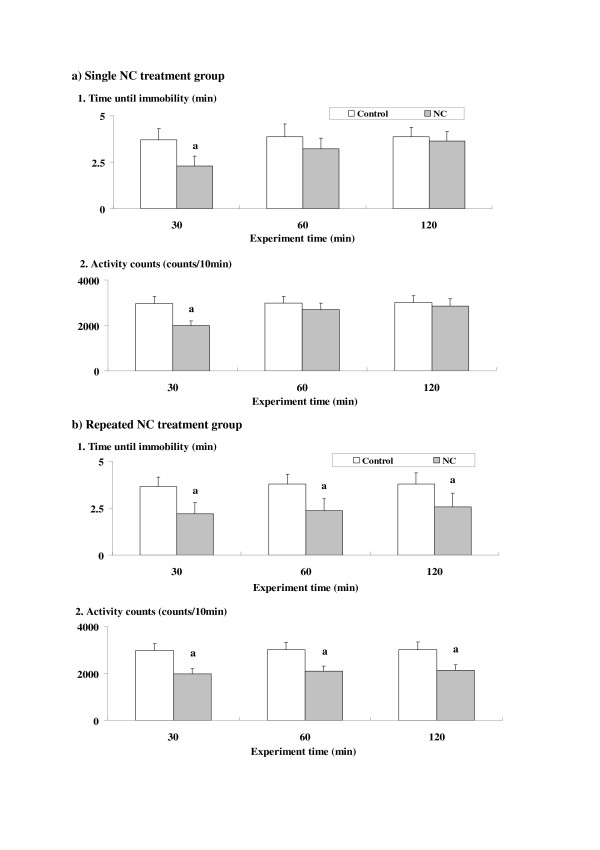
**Time courses of nicotine (NC)-induced depression-related behavioral alterations in the forced swimming test**. The parameter values at 30, 60 and 120 min time points after the NC treatment are shown for the single (a) and repeated (b) NC treatment groups. Data represent means ± SD (n = 7 for each group). The symbols for differences are the same as in the other figures.

For the single NC treatment group (Fig. 2a), at 30 min time point, depression-related alterations in swimming behaviors (significantly attenuated time until immobility and attenuated activity counts) were observed for each parameter value in the forced swimming test. However, all of the altered parameter values returned to the control levels at 60 min time point.

For the repeated NC treatment group (Fig. 2b), depression-related alterations in the swimming behaviors were observed at 30, 60, and 120 min time points.

In both single and repeated treatment groups, the ANOVA analyses revealed significant main effects of NC treatment for each parameter value [see Additional file 1]. Furthermore, significant main effects of test time and interactions between NC treatment and test time were also observed in the single treatment group, which indicated some time-dependent changes.

### Effects of anxiolytics or CBs on NC-induced anxiety-related behavioral alterations

**Figure 3:**
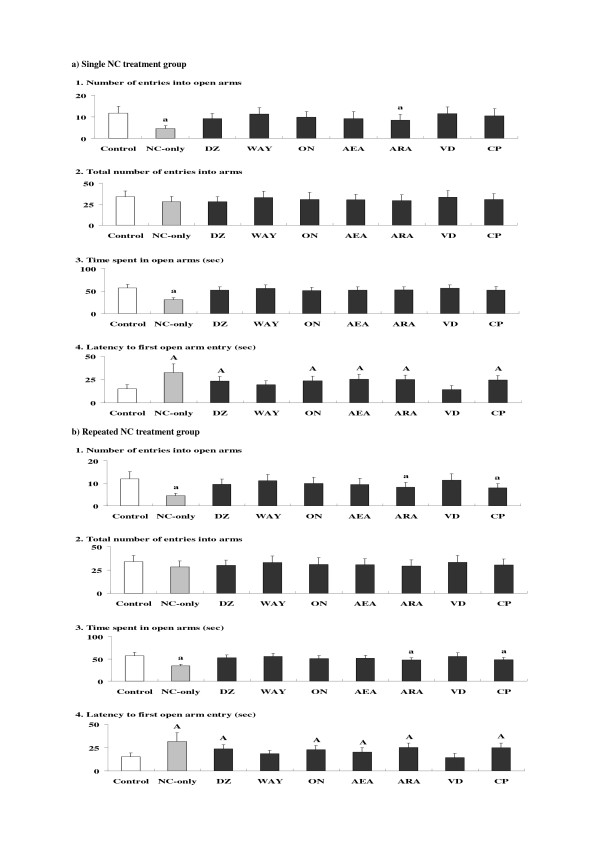
**Effects of anxiolytics or cannabinoid receptor ligands (cannabinoids: CBs) on nicotine (NC)-induced anxiety-related behavioral alterations**. The parameter values at 30 min time point for the single NC treatment group (a) and at 60 min time point for the repeated NC treatment group (b) are shown. Data represent means ± SD (n = 7 for each group). The symbols for differences are the same as the other figures. The abbreviations of the co-administered anxiolytics or CBs as demonstrated below the X-axis are noted in the text.

For the single NC treatment group (Fig. 3a), effects of anxiolytics or CBs were examined against NC-induced anxiety-related behavioral alterations observed at 30 min time point in the elevated plus-maze test. For pairwise comparisons, in the groups co-treated with the anxiolytics diazepam (DZ), WAY100135 dihydrochloride (WAY), and ondansetron hydrochloride (ON), and the CBs anandamide (arachidonylethanolamide: AEA), 2-arachidonylglycerol (ARA), virodhamine (VD), and CP55940 ((-)-cis-3- [2-Hydroxy-4-(1,1-dimethylheptyl)phenyl]-trans-4-(3-hydroxypropyl) cyclohexanol) (CP), some suppressions of the anxiety-related behavioral alterations (suppressions of the attenuated number of entries into and time spent in open arms, and/or increased latency to first open arm entry) as compared to the NC-only group were observed. In particular, in the NC-WAY and NC-VD groups, all of the parameter values returned to the control levels. On the other hand, no significant alterations were observed, either with or without the co-treatment with anxiolytics or CBs, in the total number of entries into arms.

For the repeated NC treatment group (Fig. 3b), effects of anxiolytics or CBs were examined against NC-induced anxiety-related behavioral alterations observed at 60 min time point. For pairwise comparisons, in the groups co-treated with DZ, WAY, ON, AEA, and VD, some suppressions of the anxiety-related behavioral alterations as compared to the NC-only group were observed. In particular, in the NC-WAY and NC-VD groups, all of the parameter values returned to the control levels. On the other hand, no significant alterations were observed, either with or without the co-treatment with anxiolytics or CBs, for the total number of entries into arms.

In both single and repeated treatment groups, the ANOVA analyses revealed significant main effects of NC treatment for each parameter value except for the total number of entries into arms [see Additional file 1]. Furthermore, significant main effects of anxiolytics or CBs treatment and interactions between NC and anxiolytics or CBs treatment were also observed for the time spent in open arms and the latency to first open arm entry in the single treatment group, and for each parameter value except for the total number of entries into arms in the repeated treatment group, which indicated some influences of the anxiolytics and/or CBs on the NC-induced anxiety-related behavioral alterations. However, in each anxiolytic- or CB-only group, no significant alterations in each parameter value were observed (not shown in the figure).

### Effects of anxiolytics or CBs on NC-induced depression-related behavioral alterations

**Figure 4:**
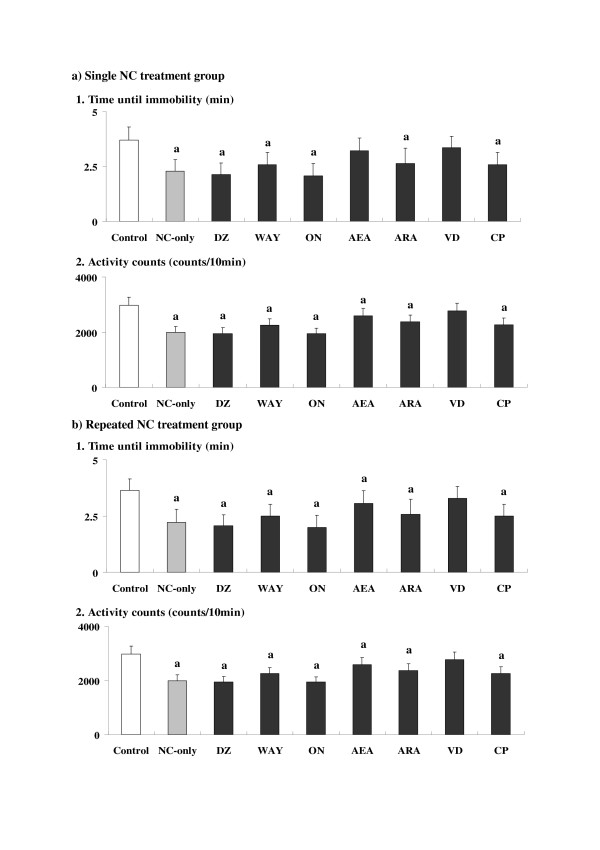
**Effects of anxiolytics or cannabinoid receptor ligands (cannabinoids: CBs) on nicotine (NC)-induced depression-related behavioral alterations**. The parameter values at 30 min time point are shown for the single (a) and repeated (b) NC treatment groups. Data represent means ± SD (n = 7 for each group). The symbols for differences are the same as the other figures. The abbreviations of the co-administered anxiolytics or CBs as demonstrated below the X-axis are noted in the text.

For the single NC treatment group (Fig. 4a), effects of anxiolytics or CBs were examined against NC-induced depression-related behavioral alterations observed at 30 min time point in the forced swimming test. For pairwise comparisons, in the groups co-treated with AEA and VD, some suppressions of the depression-related behavioral alterations (suppressions of the attenuated time until immobility and/or attenuated activity counts) as compared to the NC-only group were observed. In particular, in the NC-VD group, all of the parameter values returned to the control levels.

For the repeated NC treatment group (Fig. 4b), effects of anxiolytics or CBs were examined against NC-induced depression-related behavioral alterations observed at 30 min time point. For pairwise comparisons, only in the group co-treated with VD, significant recoveries in the depression-related behavioral alterations to the control levels were observed for each parameter value.

In both single and repeated treatment groups, the ANOVA analyses revealed significant main effects of NC and anxiolytics or CBs treatment for each parameter value [see Additional file 1]. Furthermore, significant interactions between NC and anxiolytics or CBs treatment were also observed for the activity counts, which indicated some influences of the anxiolytics and/or CBs on the NC-induced depression-related behavioral alterations. However, in each anxiolytic- or CB-only group, no significant alterations in each parameter value were observed (not shown in the figure).

## Discussion

The present experiments demonstrated chronologically overlapped occurrences of NC-induced anxiety- and depression-related behavioral alterations in both single and repeated treatment groups of mice. On the other hand, although some correlations in the underlying mechanisms could be predicted as described above, there were also some differences in the time courses between the two kinds of behavioral alterations (Figs. 1 and 2). Furthermore, an absence of antidepressant effects of many anxiolytics against the NC-induced depressed swimming behaviors was revealed (Fig. 4). With respect to the involved sites of the primary target nAChRs, the contribution of both alpha7 and alpha4beta2 subunits was suggested for the anxiogenic effects of NC [9], whereas the absence of any contribution of alpha7 subunit was demonstrated for the depressive effects of NC [31]. However, it has been reported that both anxiety- and depression-related behavioral alterations are accompanied by the increased secretion of stress hormones such as corticosteroids through the activation of the hypothalamic-pituitary-adrenal (HPA) axis [32,33]. The transient but simultaneous occurrences of anxiety- and depression-related behavioral alterations observed in the present results may be correlated with the predominant influence of the stress-related endocrine system.

As explained above, the anxiogenic effects are evaluated by an attenuated preference for open arms in the elevated plus-maze test, whereas depressive effects are evaluated by suppressed swimming behaviors (attenuated time until immobility and attenuated activity counts) in the forced swimming test. In previous studies, the opposite behavioral effects, i.e. anxiolytic and antidepressant effects, were demonstrated for NC [16,17]. Under the present conditions, only depression-related suppressed swimming behaviors were observed (Fig. 2), whereas anxiolytic effects were observed in both single and repeated treatment groups at different time points. Although the underlying mechanisms have not been elucidated sufficiently, some contributions of temporary modifications of the endogenous 5-HT and CB system have been suggested for the occurrence of anxiolytic effects [16,24]. Time-dependent modifications in the stress-related endocrine system [33] also seem to correlate with the temporary occurrence of NC-induced anxiolytic effects. Nevertheless, based on animal models of NC treatment in which anxiolytic and antidepressant effects were predominantly observed [16,17], the appearance of contrary behavioral symptoms, for example anxiogenic vs. anxiolytic symptoms, seemed to be controlled closely by the treatment conditions (i.e. dose, time after use, previous exposure to NC, etc.). For example, the antidepressant effects of NC have been reported to be affected by dose-dependent alterations in 5-HT turnover [34].

Between the single and repeated treatment groups, there were obvious differences in the time course of NC-induced anxiety- and depression-related behavioral symptoms. In the repeated treatment group, both NC-induced anxiogenic and depressive effects were prolonged as compared to the single treatment group (Figs. 1 and 2). The time of the NC-induced anxiolytic effects was also different between the single and repeated treatment groups. In addition to the modified and time-dependent activation of the HPA axis [33,35], repeated NC-induced prolonged stimulation of the sympathoadrenal system [36] may contribute to the long-lasting behavioral alterations. Furthermore, in the repeated treatment group, the antagonistic effects of some CBs observed in the single treatment group were not seen (Figs. 3 and 4). Although single NC-induced anxiety-related behavioral symptoms were partially antagonized by selective CB agonists such as ARA and CP [37,38], repeated NC, like chronic stress [39], seemed to cause an enhanced stress-related response which was not antagonized by those CB agonists. Although the induction of anxiogenic effects has been reported for CP [40], neither ARA nor CP provided any anxiogenic or anxiolytic effects in the ARA- or CP-only group, with the present doses at the selected time points (see Results section). However, anxiolytic effects were observed only in the single NC treatment group, unlike the psychostimulant treatment groups in which both ARA and CP provided anxiolytic effects in the repeated treatment groups [41]. Repeated NC treatments may specifically activate other "anxiety-related" brain receptors, in addition to enhancing stress-related responses.

With respect to the effects of anxiolytics or CBs, the absence of both antidepressant and depression-enhancing effects for the BZ- and 5-HT-related anxiolytics was demonstrated in the present study, in spite of the suggested contribution of GABAergic and 5-HT neurons to the depressive effects of NC [21,22]. Only VD, a mixed agonistic/antagonistic endocannabinoid [42], provided significant antidepressant effects in both single and repeated treatment groups. Against the anxiogenic effects of NC, VD provided significant antagonistic effects: the anxiolytic effects were as strong as WAY, an antagonist of 5-HT1A receptors for which a direct contribution to the anxiogenic effects of NC has been suggested [43,44]. Therefore, the mixed CB agonist/antagonist VD [42] was favorable for blocking the present NC-induced behavioral effects, compared with the other CBs, which functioned mainly as brain CB receptor agonists [37,38,45]. In the single treatment group, however, partial antagonistic effects of AEA restricted to the activity count parameter were provided against the depressed swimming behaviors. AEA and the strong selective CB agonists ARA and CP partially attenuated the single NC-induced anxiety-related behavioral alterations in the elevated plus-maze test. Although the detailed characteristics of each CB (e.g. indirect target receptors, etc.) have not been elucidated, the partial antagonistic effects of AEA against the depressed swimming behaviors may be correlated with the presence of specific effects and/or targets of AEA as previously suggested [46].

## Conclusion

The present study demonstrated a chronological overlap for the anxiogenic and depressive effects of NC. A prolongation of these behavioral effects caused by repeated administrations and early transient anxiolytic effects seemed to lead to the reinforcement of habitual use of NC, similar to other addictive drugs such as harmful psychostimulants.

Among anxiolytics and CBs, the endogenous CB VD, which is known to act as a mixed agonist/antagonist on brain CB receptors, attenuated both anxiogenic and depressive effects of NC. This supports the contribution of brain CB receptors to NC-induced anxiety- and depression-related behavioral symptoms. Furthermore, one can predict that the role of VD as a mixed agonist/antagonist on brain CB receptors contributed to its double validity as both an anxiolytic and an antidepressant.

## Methods

### Animals

Male ICR mice (60–90 days old) (Shizuoka Laboratory Animal Center, Hamamatsu, Japan) were housed in a forced-air facility, which was maintained at 23°C and 50 % relative humidity, with a 12 h/12 h light/dark cycle [47,48]. The mice were kept separately in single transparent cages measuring 23.5 × 16.5 × 12 cm, and were allowed water and rodent chow ad libitum [47,48]. The experiments described in this report were conducted in accordance with the "Guidelines for Animal Experiments" of our institution (1988), which are based on the National Institutes of Health Guide for Care and Use of Laboratory Animals, and any pain experienced by the mice was minimized. Each experimental group contained 7 mice.

### Drug treatments

Doses of nicotine (NC) (Nacalai Tesque, Inc., Kyoto, Japan) were determined based on preliminary experiments and previous studies examining its behavioral effects in single and repeated treatment groups [49,50]. After the preliminary experiments, the doses of NC which caused anxiogenic and depressive effects most predominantly were selected. For the single treatment group, a single subcutaneous (s.c.) dose of 0.5 mg/kg was administered. For the repeated treatment group, a s.c. dose of 0.3 mg/kg was administered daily for 4 days.

The anxiolytics except for CBs were selected among the drugs which are known to act as anxiolytics and have been demonstrated to attenuate the effects of NC in preliminary experiments and previous studies [7,9,51-53]. The drugs selected were the BZ agonist diazepam (DZ) [51,53] purchased from Astellas Pharma Inc. (Tokyo, Japan), the 5-HT1A antagonist WAY100135 dihydrochloride (WAY) [9] purchased from Tocris Cookson Inc. (Ellisville, Missouri, USA), and the 5-HT3 antagonist ondansetron hydrochloride (ON) [7,52] purchased from GlaxoSmithKline Inc. (London, UK).

As CBs, the endogenous CBs anandamide (arachidonylethanolamide: AEA) [45], 2-arachidonylglycerol (ARA) [38], and virodhamine (VD) [42], and the potent and synthetic agonist CP 55940 ((-)-cis-3- [2-Hydroxy-4-(1,1-dimethylheptyl)phenyl]- trans-4-(3-hydroxypropyl)cyclohexanol) (CP) [37], all purchased from Tocris Cookson Inc., were selected. With respect to these CBs, the anxiolytic effects except for those provided by AEA [29] have not been examined sufficiently. However, based on a study examining the psychostimulant-induced anxiety-related behavioral symptoms [41], the CBs which provided some anxiolytic effects against the psychostimulant-induced symptoms were selected.

With respect to the dose of the anxiolytics and CBs, a single dose was selected after preliminary experiments examining a broad range of concentrations. For each drug, data were collected for the dose which provided no anxiety- and depression-related behavioral alterations by itself at the selected time points, but which antagonized the NC-induced anxiety-related behavioral symptoms most effectively. For the single treatment group, an intraperitoneal (i.p.) injection of 5 mg/kg for DZ, 1 mg/kg for WAY, 0.01 mg/kg for ON, 10 mg/kg for each endocannabinoid, or 2.5 mg/kg for CP was performed before the NC treatment. For the repeated treatment group, an i.p. injection of the same dose of each anxiolytic and CB was performed repeatedly before each NC treatment. Except for the CBs, the anxiolytics were administered 15 min before each NC treatment [7,9,51-53], whereas the CBs were administered 60 min before each NC [54], based on previous studies and preliminary experiments.

NC was dissolved in saline to a volume of 5 ml/kg for administration. Since the CBs and WAY are not soluble in water, they were initially dissolved in dimethylsulphoxide (DMSO) (Nacalai Tesque Inc.) to one-third of the total volume, and then diluted in distilled water to a volume of 5 ml/kg. Since AEA, ARA, and VD were provided in ethanol solutions (Tocris Cookson Inc.), the ethanol was evaporated immediately before use under nitrogen gas, and the residues were initially dissolved in DMSO to one-third of the total volume, and then diluted in distilled water to a volume of 5 ml/kg [55,56]. The other anxiolytics were also dissolved in DMSO to one-third of the total volume, and then diluted in distilled water to a volume of 5 ml/kg. In the NC-only group, even in the time course study of the NC-only treatment, a mixed vehicle solution of DMSO and distilled water at the same ratio as the solutions of anxiolytics or CBs was injected instead of the anxiolytics or CBs, 15 min before each NC administration. In the anxiolytic- or CB-only groups, the same volumes of saline vehicle were injected instead of NC. In the control group without any drug treatment (control group), a mixed vehicle solution of DMSO and distilled water at the same ratio as the anxiolytic or CB solutions was injected instead of the anxiolytics or CBs, and then the same volume of saline vehicle was injected instead of NC. The drug administration and each experimental session were performed between 15 and 19 hrs light cycle.

### Evaluation of anxiety-related behavioral alterations (elevated plus-maze test)

Based on previous studies [41,57], alterations in anxiety-related behaviors were examined by the elevated plus-maze test using an apparatus that consisted of two opposite open arms 50 × 10 cm (length and width) and two closed arms 50 × 10 × 30 cm (length, width, and height). As parameters for the test (5 min test periods), the number of entries into open arms, the total number of entries into arms, the time spent in open arms (sec), and the latency to first open arm entry (sec) were evaluated. In the time course study, evaluations of these parameters were performed at 30, 60, and 120 min time points after the NC treatment. The parameters were evaluated after placing each mouse diagonally in the center of the maze, facing both the open and closed arms.

### Evaluation of depression-related behavioral alterations (forced swimming test)

Based on previous studies [48,58], alterations in depression-related behaviors were examined by the forced swimming test using a glass cylinder apparatus 33 cm in height and 18 cm in diameter containing 14 cm of water at 21–23°C. As parameters for the test, the time until immobility (the time after when only modest swimming behaviors necessary to avoid drowning were observed) and the activity counts for 10 min yielded by swimming behaviors were evaluated. In the time course study, evaluations of these parameters were performed at 30, 60, and 120 min time points after the NC treatment. The activity was counted using the activity-measuring and recording system Supermex-CompACT AMS instrument (Muromachi Kikai Co. Ltd., Tokyo, Japan) by placing the sensor of the instrument over the cylinder at a distance of 20 cm from the water.

### Statistical analysis

The data obtained were subjected to two-way analysis of variance (ANOVA) for both single and repeated treatment groups [41,59]. In the experiments of the time course of the NC-induced behavioral alterations, a 2 (NC versus vehicle) × 3 (30 min, 60 min versus 120 min) factorial design was used for the factors NC treatment × test time. In the experiments of the effects of the anxiolytics or CBs, a 2 (NC versus vehicle) × 8 (DZ, WAY, ON, AEA, ARA, VD, CP versus vehicle) factorial design was used for the factors NC treatment × treatment of each anxiolytic or CB drug. The results from ANOVA analyses are summarized in the table [see Additional file 1]. For pairwise comparisons, post-hoc Bonferroni tests were performed [41,59]. All of the comparisons were performed using statistical software packages ('Excel Statistics' from Social Survey Research Information Co. Ltd. Inc., Tokyo, Japan). Unless otherwise noted, P values less than 0.05 were considered to be statistically significant.

## Authors' contributions

TH designed the study, carried out all experiments and statistical analyses, and prepared the manuscript.

## Supplementary Material

Additional file 1 Summary of statistical analyses. F values with the degrees of freedom are shown. Significant effects and interactions are noted: * P < 0.05, ** P < 0.01, ***P < 0.001. Click here for file
