# Supplementary material for: Chronologically overlapping occurrences of nicotine-induced anxiety- and depression-related behavioral symptoms: effects of anxiolytic and cannabinoid drugs
Source: BMC Neurosci. 2007 Sep 18;8:76. doi: 10.1186/1471-2202-8-76 (PMC2075518; doi:10.1186/1471-2202-8-76)
Supplement: Additional file 1 — Summary of statistical analyses. F values with the degrees of freedom are shown. Significant effects and interactions are noted: * P < 0.05, ** P < 0.01, ***P < 0.001. [file 1471-2202-8-76-S1.doc]

a) Parameters in the time course of single NC-induced anxiety-related behavioral alterations (Fig. 1a)

|  | Number of entries into open arms | Total number of entries into arms | Time spent in open arms | Latency to first open arm entry |
| --- | --- | --- | --- | --- |
| NC treatment | F(1, 36)=11.06** | F(1, 36)=13.81*** | F(1, 36)=6.89* | F(1, 36)=6.74* |
| Test time | F(2, 36)=4.84* | F(2, 36)=1.03 | F(2, 36)=80.56*** | F(2, 36)=11.30*** |
| NC treatment × test time interaction | F(2, 36)=4.97* | F(2, 36)=0.90 | F(2, 36)=80.85*** | F(2, 36)=12.03*** |

b) Parameters in the time course of repeated NC-induced anxiety-related behavioral alterations (Fig. 1b)

|  | Number of entries into open arms | Total number of entries into arms | Time spent in open arms | Latency to first open arm entry |
| --- | --- | --- | --- | --- |
| NC treatment | F(1, 36)=66.36*** | F(1, 36)=16.21*** | F(1, 36)=34.33*** | F(1, 36)=25.12*** |
| Test time | F(2, 36)=1.63 | F(2, 36)=1.58 | F(2, 36)=15.04*** | F(2, 36)=14.27*** |
| NC treatment × test time interaction | F(2, 36)=1.77 | F(2, 36)=1.69 | F(2, 36)=15.83*** | F(2, 36)=13.91*** |

c) Parameters in the time course of single NC-induced depression-related behavioral alterations (Fig. 2a)

|  | Time until immobility | Activity counts |
| --- | --- | --- |
| NC treatment | F(1, 36)=15.84*** | F(1, 36)=23.29*** |
| Test time | F(2, 36)=5.43** | F(2, 36)=7.88** |
| NC treatment × test time interaction | F(2, 36)=3.45* | F(2, 36)=7.16** |

d) Parameters in the time course of repeated NC-induced depression-related behavioral alterations (Fig. 2b)

|  | Time until immobility | Activity counts |
| --- | --- | --- |
| NC treatment | F(1, 36)=45.76*** | F(1, 36)=104.34*** |
| Test time | F(2, 36)=0.52 | F(2, 36)=0.34 |
| NC treatment × test time interaction | F(2, 36)=0.13 | F(2, 36)=0.10 |

e) Parameters in the effects of anxiolytics or CBs on single NC-induced anxiety-related behavioral alterations (Fig. 3a)

|  | Number of entries into open arms | Total number of entries into arms | Time spent in open arms | Latency to first open arm entry |
| --- | --- | --- | --- | --- |
| NC treatment | F(1, 96)=11.03** | F(1, 96)=1.43 | F(1, 96)=12.59*** | F(1, 96)=41.46*** |
| Anxiolytics or CBs treatment | F(7, 96)=1.74 | F(7, 96)=0.65 | F(7, 96)=3.49** | F(7, 96)=4.06*** |
| NC × anxiolytics or CBs treatment interaction | F(7, 96)=1.93 | F(7, 96)=0.20 | F(7, 96)=4.26*** | F(7, 96)=2.93** |

f) Parameters in the effects of anxiolytics or CBs on repeated NC-induced anxiety-related behavioral alterations (Fig. 3b)

|  | Number of entries into open arms | Total number of entries into arms | Time spent in open arms | Latency to first open arm entry |
| --- | --- | --- | --- | --- |
| NC treatment | F(1, 96)=16.07*** | F(1, 96)=2.13 | F(1, 96)=19.87*** | F(1, 96)=39.69*** |
| Anxiolytics or CBs treatment | F(7, 96)=2.70* | F(7, 96)=0.51 | F(7, 96)=4.12*** | F(7, 96)=4.49*** |
| NC × anxiolytics or CBs treatment interaction | F(7, 96)=3.08** | F(7, 96)=0.17 | F(7, 96)=3.87*** | F(7, 96)=3.28** |

g) Parameters in the effects of anxiolytics or CBs on single NC-induced depression-related behavioral alterations (Fig. 4a)

|  | Time until immobility | Activity counts |
| --- | --- | --- |
| NC treatment | F(1, 96)=50.10*** | F(1, 96)=149.09*** |
| Anxiolytics or CBs treatment | F(7, 96)=2.63* | F(7, 96)=6.12*** |
| NC × anxiolytics or CBs treatment interaction | F(7, 96)=2.03 | F(7, 96)=3.67** |

h) Parameters in the effects of anxiolytics or CBs on repeated NC-induced depression-related behavioral alterations (Fig. 4b)

|  | Time until immobility | Activity counts |
| --- | --- | --- |
| NC treatment | F(1, 96)=74.98*** | F(1, 96)=160.65*** |
| Anxiolytics or CBs treatment | F(7, 96)=3.35** | F(7, 96)=6.75*** |
| NC × anxiolytics or CBs treatment interaction | F(7, 96)=1.12 | F(7, 96)=3.39** |
